# Religiousness, sexual orientation, and depression among emerging adults in U.S. higher education: Findings from the Healthy Minds Study

**DOI:** 10.1371/journal.pmen.0000004

**Published:** 2025-03-26

**Authors:** Hans Oh, G. Tyler Lefevor, Edward B. Davis, Anna Zhu, Yaofang Hu, Trevor A. Pickering, Ai Koyanagi, Lee Smith

**Affiliations:** 1 Suzanne Dworak Peck School of Social Work, University of Southern California, Los Angeles, California, United States of America; 2 Department of Psychology, College of Education and Human Services, Utah State University, Utah, United States of America; 3 Department of Psychology, Wheaton College, Wheaton, Illinois, United States of America; 4 Southern Methodist University, Dallas, Texas, United States of America; 5 Research and Development Unit, Parc Sanitari Sant Joan de Déu, CIBERSAM, ISCIII, Sant Boi de Llobregat, Barcelona, Spain; 6 Centre for Health, Performance and Wellbeing, Anglia Ruskin University, Cambridge, United Kingdom; The University of Waikato, NEW ZEALAND

## Abstract

Religiousness has long been found beneficial for mental health, although its protective effects may be attenuated for sexual minorities. We sought to examine the associations between religiousness and depression and whether these associations were moderated by religious affiliation and/or sexual orientation. We analyzed the dataset (*N* = 103,161 undergraduate and graduate students) from the Healthy Minds Study (2020-2021), which was an online survey administered at 140 higher education institutions across the United States. We used multivariable logistic regression to examine the associations between religiousness (religious affiliation and importance) and depression, adjusting for age, gender identity, and race/ethnicity. We tested for effects moderated by religious affiliation and/or sexual orientation. Associations between religious importance and depression varied across sexual minority groups (gay/lesbian, bisexual, or other) and religious affiliations, although the differences across sexual minority groups were only statistically significant among Catholic students. Broadly, among Christian students, higher religious importance was associated with lower odds of depression, but this protective association was only evident among heterosexual students (and not among sexual minority students). Higher religious importance was also associated with lower odds of depression among Muslim students, but again this effect was only present among heterosexual students. Among students who identified as Hindu or Catholic, religious importance was associated with lower odds of depression among students who identified as heterosexual or queer/questioning/other. Among those who identified as Buddhist or Mormon, religious importance was associated with greater odds of depression for bisexual students. In conclusion, religiousness was associated with lower odds of depression for young adults generally. This association was moderated by sexual orientation, showing often weaker or non-significant effects among sexual minorities, depending on sexual orientation and religious affiliation.

## Introduction

According to the National Surveys on Drug Use and Health, the 12-month prevalence of major depressive episodes increased from 8.7% in 2005 to 11.3% in 2014 among adolescents, and increased from 8.8% to 9.6% among young adults in the United States [1]. In higher education, emerging adults encounter a host of stressors while navigating new and challenging social environments [2–4]. Moreover, emerging adults embark on a journey of self-discovery, re-examining their assumptions about the world and their places within it. These socio-environmental changes and exposures to new experiences can sometimes make them vulnerable to a host of mental health problems, including depression [5,6]. Depression has been related to worse academic performance, greater impairment, and greater risk for suicide [7,8]. Studies suggest that depression increased during the COVID-19 pandemic [9] and disproportionately impacted students across U.S. higher education institutions [10,11]. For example, in a non-probability survey of over 100,000 students in U.S. higher education institutions during the first year of the COVID-19 pandemic, around one-in-five reported *moderately severe* to *severe* depression symptoms [11]. Thus, it is imperative to study the risk and protective factors for depression in this population.

One well-established protective factor for mental health is religiousness [12]. Religiousness is defined as the search for the sacred in the context of culturally sanctioned rituals, belief/value systems, and institutions [13]. Religiousness is a multi-dimensional construct and aspects of religiousness have long been identified as beneficial for mental health for many people [14]. Dimensions of religiousness include the sacred beliefs that people hold, the bonds that connect people to the sacred, the behaviors that people exhibit that are aligned with a belief system, and a sense of belonging in a religious tradition or community [15]. The ways in which religion impacts depression may be complex, but putative main pathways include shaping schema about one’s circumstances or shaping one’s views about the world [16,17], creating opportunities to build relationships with co-religionists and receiving social support from them [18,19], and providing a framework for coping, such as through rituals and religious practices (e.g., prayer) [20]. Despite its multidimensional nature, religiousness is often measured in surveys using religious affiliation or religious importance as proxies [21].

Several meta-analyses have documented a positive relationship between religiousness and better mental health [22,23], including a meta-analysis focused specifically on adolescents and emerging adults [21]. Together, these analyses suggest that when individuals report experiencing higher levels of religiousness, they also report lower psychological distress [24], depression [22,23], anxiety [25], and suicidal thoughts and behaviors [26]. The existing literature on the protective effects of religiousness have highlighted several potential pathways [17]. However, specific aspects of religiousness have not been universally beneficial for people across all social contexts.

Sexual minorities often have a more complicated relationship with religion than their heterosexual counterparts do. Sexual minorities are individuals who report a significant degree of same-sex sexual attraction, engage in same-sex sexual behavior, and/or identify as lesbian, gay, bisexual, or queer/questioning [27]. Although an estimated 50-60% of sexual minorities are religious (59%; [28]), many sexual minorities report experiences of discrimination from religious people and places [29,30], likely due to oppressively experienced religious beliefs or practices (e.g., the belief that homosexuality is a sin and the practice of excluding sexual minorities from religious leadership positions). This discrimination may make it more difficult for sexual minorities to access the psychological benefits of religiousness that heterosexual individuals may more readily experience [31].

These difficulties are evident in the empirically observed relationship between religiousness and mental health among sexual minorities [29,32–35]. A recent meta-analysis found that religiousness was positively related to mental health for sexual minorities [36]; however, this relationship was much weaker (*r* =.05) for sexual minorities, relative to what is typically observed among heterosexual individuals (*r* =.15). Further, approximately half of the studies included in this analysis reported a *negative* relationship between religiousness and mental health among sexual minorities, suggesting considerable variation in how sexual minorities experience and express their religiousness. One study of college students found that increased importance of religion was associated with greater odds of recent suicidal ideation for sexual minority students, and lesbian/gay students who viewed religion as *very important* had greater odds of recent suicidal ideation and lifetime suicide attempts when compared with heterosexual students. Upon disaggregating the sexual minority category, the authors found that, across all levels of religious importance, bisexual and questioning sexual orientations were associated with increased risk of recent suicidal ideation, recent suicide attempts, and lifetime attempts, but associations were strongest among those who reported that religion was *very important* to them [35].

Whether religion proves to be a blessing or a risk factor for sexual minorities may largely depend on the belief systems and practices that organize their religious lives. For example, sexual minorities who have an internal conflict between their sexuality and their religious beliefs may be at more risk for mental health problems, as evidenced by the harmful psychological effects of religious sexual orientation change efforts [37]. Some have noted that while religiousness may be positively related to suicidal ideation among gay and lesbian students, much depends on one’s sense of belonging and the coherence between one’s religious beliefs and one’s sexual identity [38]. Further, there appears to be emerging evidence that sexual minority students attending religious universities may not necessarily exhibit particularly high risk of suicidal ideation or anxiety when compared with sexual minority students at non-religious universities [39].

Younger generations are increasingly identifying as gay, lesbian, bisexual, and queer (around 16% of Gen Z individuals; [40]), and around 40% of LGBTQ+ adults aged 18-34 report being religious [41]. As such, more research is needed to confirm whether the association between religiousness and mental health depends on sexual minority status in different subpopulations. In this study, we contribute to the scientific literature by analyzing data from a large sample of emerging-adult undergraduate and graduate students enrolled in one of 140 U.S. higher education institutions. We examined the associations between religiousness and depression, using two different measures of religiousness (religious affiliation and religious importance), testing whether the associations were different across sexual orientations and religious affiliations*.* In doing so, we build on the existing literature by exploring the associations in a large and religiously, culturally, and sexually diverse sample of emerging adults.

## Methods

### Sample

The Healthy Minds Study is a non-probability online survey administered to undergraduate and graduate students in 140 U.S. higher education institutions [42]. The survey was administered online between September 2020 and June 2021. Given the heightened prevalence both of depression and sexual-minority identification among younger generations [11,43], we limited the sample to emerging adults (aged 18-29). We used complete-case analysis, and ran sensitivity analyses using multiple imputation, which are available upon request. The response rate for the survey was 14%. To account for non-response, we used sample probability weights (based on administrative data at each institution; [42]. We excluded individuals who were missing data on any of the variables of interest, resulting in an analytic sample of 103,161.

### Ethics statement

The HMS was approved by the Institutional Review Board, Advarra (IRB number: Pro00028565), as well as the Institutional Review Boards at all participating campuses, which collectively provided ethical oversight. All respondents provided informed consent. The secondary analysis presented in this study was deemed exempt under the approval of University of Southern California (UP-22-00068). The HMS data are available upon request at: https://healthymindsnetwork.org/hms/.

### Measures

#### Depression (outcome).

Depression was measured using the Patient Health Questionnaire–9 (PHQ-9; [44], which assesses the frequency of depression symptoms over the past two weeks. The items were summed into a scale (range: 0-27), which was dichotomized (cut-off: 10; [45]) to indicate the presence of moderately severe or severe depression. The PHQ-9 has been validated in ethno-racially diverse student populations in the US. The scale demonstrated excellent reliability in this sample (α = .90).

#### Religiousness (predictors).

Religiousness was measured using two items. One assessed religious affiliation, and the other assessed religious importance [15,46].

#### Religious affiliation.

Respondents were provided a checklist containing the following religious affiliations: Agnostic, Atheist, Buddhist, Catholic, Protestant, Hindu, Jewish, Muslim, Mormon, Other, and No Preference. Individuals who indicated ‘Other’ were then asked to specify their affiliations in a free-response field. We manually recoded the ‘Other’ responses in accordance with the religious affiliation classification system used by the Pew Research Center ([47]), and thus created additional categories, including Other Christian (e.g., United Methodist, Southern Baptist, Seventh-Day Adventist, Russian Orthodox, Roman Catholic, Presbyterian, Pentecostal), Wiccan/Pagan (e.g., Norse Pagan, Witchcraft), Nothing in Particular, Spiritual But Not Religious, Other World Religion (e.g., Sikhism, Shintoism, Shamanism, Punjabi, Jainism, Daoism, Bahai), Other Faith (e.g., Unitarian Universalist, Theism, Secular Humanism, Satanism, New Age Spiritualism, Native American Spiritualism), Mixed/Blended Faith (e.g., Quaker Pagan, Polytheism, Pantheism), and I Don’t Know (e.g., still figuring it out, questioning, prefer not to say; these individuals were omitted from the study). We coded created a Multiple Religion category that includes individuals who selected more than one affiliation (note that this also includes individuals who endorsed both Atheism *and* Agnosticism). We coded broader categories as follows: Unaffiliated (Atheism, Agnosticism, Nothing in Particular, No Preference), Christian Religious, Non-Christian Religious, Multiple Religions, and I Don’t Know.

#### Religious importance.

A second religious dimension was religious importance, using a single item: “How important is religion in your life?” Respondents could answer: *very important*, *important*, *neutral*, *unimportant*, and *very unimportant*. This item was coded 1 (*very unimportant*) through 5 (*very important*), so that higher scores indicated higher religious importance.

#### Sexual orientation (moderator).

Respondents were asked: “How would you describe your sexual orientation?” Respondents could answer: *Heterosexual*, *Lesbian*, *Gay*, *Bisexual*, *Queer*, *Questioning*, or *Other (self-identified)*. We coded the sexual orientation variable categorically to reflect the following four sexual orientation groups: heterosexual, lesbian/gay, bisexual, and queer/questioning/other. Lesbia/gay, bisexual, and queer/questioning/other were considered 'sexual minority groups'.

#### Sociodemographic characteristics (covariates).

Respondents also self-reported gender identity (man [cisgender], woman [cisgender], transgender/nonbinary/other), age (continuous), and race/ethnicity (White, Black, Latinx/Hispanic, Asian American/Pacific Islander, multiracial, other).

### Analysis

In the Supplemental Materials, we provide the unweighted descriptive statistics for the analytic sample, including religious affiliations, religious importance, and the prevalence of depression, stratified by sexual orientation identity. We disaggregated the sexual minority group into gay/lesbian, bisexual, and other (queer, questioning, other/self-identified) to provide more nuanced picture of sexual orientation identity. For our main analyses, we applied survey weights to account for non-response. These sample probability weights were created by the HMS investigators using administrative data on full student populations at each participating institution with respect to gender, race/ethnicity, Grade Point Average, and academic level (ungraduated, graduate). Standard errors were clustered by institution.

With these survey weights, we ran multivariable logistic regression analyses to examine the associations between broad categories of religious affiliation (Christian, non-Christian, multiple religions, I don’t know) and depression, and then included an interaction term (religious affiliation x sexual minority status). We also examined the associations between religious importance and depression in the entire analytic sample and then stratified by religious affiliation. We treated religious importance as a continuous variable to allow us to more easily interpret the interactions between religious importance and a four-category moderator (sexual orientation) across twelve religious affiliations. We ran overall interaction likelihood ratio tests within each religious affiliation to determine if there were significant differences in the effect of religious importance across sexual minority categories. An alpha of 0.05 was used for distinguishing potential moderation effects. We did not examine interactions with religious groups that made up less than 1% of the sample (i.e., Other World Religion, Other Faith, Wiccan/Pagan, Nothing in Particular, Spiritual but Not Religious, and Mixed/Blended Faith); these categories were not large enough to analyze and were heterogeneous, making interpretation difficult. Individuals who stated ‘I don’t know’ in response to the religious affiliation item were not included in the analyses.

All models were adjusted for age, gender identity, race/ethnicity. We present results as odds ratios with 95% confidence intervals, and we interpret the odds (and not probabilities) acknowledging that odds ratios can become less accurate when used with a relatively common outcome like moderately severe to severe depression, which was reported by approximately 42% of the sample [48–51]. We ran sensitivity analyses using multiple imputation; however, findings overall did not change, and we therefore present the complete case analyses. We performed all statistical analyses using R.

## Results

### Descriptive statistics

Table 1 provides unweighted descriptive statistics for the analytic sample. Most of the sample was heterosexual (74.4%), with approximately 3.5% identifying as gay/lesbian; 11.4% identifying as bisexual; and 10.6% identifying queer, questioning, or other (self-identified). Consistent with estimates from the general population, the bisexual group was the largest among the sexual minority groups. Approximately two-thirds of the sample was religiously affiliated, with more than half the entire sample being affiliated with Christianity. Around 8% of the sample was affiliated with a non-Christian religion. Religious affiliation was higher among heterosexual students than sexual minority students, though around half of sexual minority students were religiously affiliated. S1 Fig shows the composition of sexual orientation within each religious affiliation. Over 42% of the sample met the criteria for clinical depression (i.e., a PHQ-9 score > 9, moderate to severe), with a significantly higher prevalence among gay/lesbian students (53.8%), bisexual students (64.5%), and queer/questioning/other students (62.8%), relative to heterosexual students (36.8%). Depression prevalence was the lowest among Christian religious affiliation (36.6%), and the highest among people with multiple religious affiliations (53.3%), followed by Unaffiliated (49.6%) (S2 Fig; S3 Fig). Approximately 41.5% reported that religion was *important* or *very important* to them, with a higher percentage among heterosexual students (48%) than among gay/lesbian (19.4%), bisexual (21.7%), and queer/questioning/other students (24.2%) (S4 Fig).

**Table 1:** Sample characteristics.

	Self-reported sexual orientation identity				
	Heterosexual	Gay/Lesbian	Bisexual	Other	*p*-value
	*N* = 76,773 (74.4%)	*N* = 3,661 (3.5%)	*N* = 11,783 (11.4%)	*N* = 10,954 (10.6%)	
Religious affiliation					
No preference	9,846 (12.8%)	732 (20.0%)	2,156 (18.3%)	1,871 (17.1%)	<.001
Atheist	4,500 (5.9%)	533 (14.6%)	1,576 (13.4%)	1,243 (11.3%)	
Agnostic	6,437 (8.4%)	665 (18.2%)	2,415 (20.5%)	1,927 (17.6%)	
Nothing in particular	119 (0.2%)	21 (0.6%)	20 (0.2%)	66 (0.6%)	
Catholic	19,587 (25.5%)	499 (13.6%)	1,495 (12.7%)	1,237 (11.3%)	
Protestant	18,919 (24.6%)	408 (11.1%)	1,285 (10.9%)	1,193 (10.9%)	
Other Christian	4,406 (5.7%)	89 (2.4%)	362 (3.1%)	403 (3.7%)	
Mormon	2,100 (2.7%)	22 (0.6%)	97 (0.8%)	105 (1.0%)	
Jewish	1,644 (2.1%)	120 (3.3%)	232 (2.0%)	300 (2.7%)	
Muslim	1,969 (2.6%)	15 (0.4%)	106 (0.9%)	186 (1.7%)	
Buddhist	1,003 (1.3%)	51 (1.4%)	176 (1.5%)	172 (1.6%)	
Hindu	1,443 (1.9%)	13 (0.4%)	82 (0.7%)	86 (0.8%)	
Other world religion	246 (0.3%)	7 (0.2%)	37 (0.3%)	44 (0.4%)	
Spiritual but not religious	334 (0.4%)	38 (1.0%)	199 (1.7%)	204 (1.9%)	
Other faith	224 (0.3%)	22 (0.6%)	96 (0.8%)	119 (1.1%)	
Mixed/blended faith	82 (0.1%)	11 (0.3%)	60 (0.5%)	66 (0.6%)	
Wiccan/Pagan	78 (0.1%)	48 (1.3%)	233 (2.0%)	214 (2.0%)	
Multiple religions	3,836 (5.0%)	367 (10.0%)	1,156 (9.8%)	1,518 (13.9%)	
Depression					
No	48,557 (63.2%)	1,690 (46.2%)	4,184 (35.5%)	4,079 (37.2%)	<.001
Yes	28,216 (36.8%)	1,971 (53.8%)	7,599 (64.5%)	6,875 (62.8%)	
Religious importance					
Very unimportant	10,878 (14.2%)	1,213 (33.1%)	3,554 (30.2%)	3,100 (28.3%)	<.001
Unimportant	10,320 (13.4%)	813 (22.2%)	2,680 (22.7%)	2,318 (21.2%)	
Neutral	18,703 (24.4%)	926 (25.3%)	2,991 (25.4%)	2,889 (26.4%)	
Important	17,791 (23.2%)	489 (13.4%)	1,834 (15.6%)	1,764 (16.1%)	
Very important	19,081 (24.9%)	220 (6.0%)	724 (6.1%)	883 (8.1%)	
Age	21.34 (2.83)	21.34 (2.87)	21.05 (2.62)	21.02 (2.70)	<.001
Gender identity					
Man	23,724 (30.9%)	1,603 (43.8%)	1,493 (12.7%)	1,525 (13.9%)	<.001
Woman	52,912 (68.9%)	1,633 (44.6%)	9,445 (80.2%)	7,344 (67.0%)	
Queer/non-binary/other	137 (0.2%)	425 (11.6%)	845 (7.2%)	2,085 (19.0%)	
Race/ethnicity					
White	47,022 (61.2%)	2,330 (63.6%)	7,502 (63.7%)	6,641 (60.6%)	<.001
Asian/Pacific Islander	9,966 (13.0%)	314 (8.6%)	949 (8.1%)	1,107 (10.1%)	
Black/African American	6,627 (8.6%)	314 (8.6%)	903 (7.7%)	922 (8.4%)	
Hispanic/Latinx	5,227 (6.8%)	262 (7.2%)	856 (7.3%)	704 (6.4%)	
Two or more/multiracial /multiethnic	6,573 (8.6%)	401 (11.0%)	1,452 (12.3%)	1,431 (13.1%)	
Other	1,358 (1.8%)	40 (1.1%)	121 (1.0%)	149 (1.4%)	

*Note*. *N* (unweighted %); *p*-values reflect results of chi-square tests.

### Multivariable regression analyses

The prevalence of clinical depression varied across religious affiliations (S2 Fig), with the prevalence being highest among those who identified as Wiccan/Pagan. Students who identified as Hindu had the lowest prevalence. In Table 2, we display that, adjusting for age, gender, and race/ethnicity, Christian religious affiliation was associated with significantly lower odds of depression (aOR: 0.70; 95% CI: 0.66-0.75), when compared with religiously unaffiliated students. Non-Christian religious affiliation was not significantly associated with depression, but having multiple religious affiliations was associated with greater odds of depression (aOR: 1.09; 95% CI: 1.01-1.18).

**Table 2:** Multivariable logistic regression models showing associations between religious affiliation and depression over the past two weeks, Healthy Minds Survey 2020-2021 (*N* = 103,161).

Religious affiliation (and interaction by sexual minority status)	aOR (95% CI)	*p*-value	aOR (95% CI)	*p*-value
Religiously unaffiliated	1.00		1.00	
Christian	**0.70 (0.66, 0.75)**	**<.001**	0.68 (0.64, 0.73)	<.001
Non-Christian religion	0.97 (0.9, 1.05)	.502	0.90 (0.82, 1.00)	.040
Multiple religions	**1.09 (1.01, 1.18)**	**.026**	1.07 (0.97, 1.19)	.165
Don’t know	0.86 (0.54, 1.38)	.528	0.77 (0.42, 1.40)	.387
Religiously unaffiliated x sexual minority			1.00	
Christian x sexual minority			**1.13 (1.00, 1.27)**	**.043**
Non-Christian religion x sexual minority			**1.25 (1.05, 1.50)**	**.012**
Multiple religions x sexual minority			1.06 (0.92, 1.22)	.454
Don’t know x sexual minority			1.34 (0.53, 4.12)	.449

*Note*. Odds ratios were adjusted for age, gender, and race/ethnicity. P<0.05 indicated in bold

When examining interactions, we found that sexual orientation moderated the association between depression and both Christian and non-Christian religious affiliations, such that religious affiliation was less protective for sexual minority students than it was for heterosexual students. We present the associations between religious affiliation and depression, stratified by sexual orientation [S1 Table; S2 Table]. Among sexual minority students, non-Christian religious affiliation was associated with greater odds of depression.

**Fig 1:**
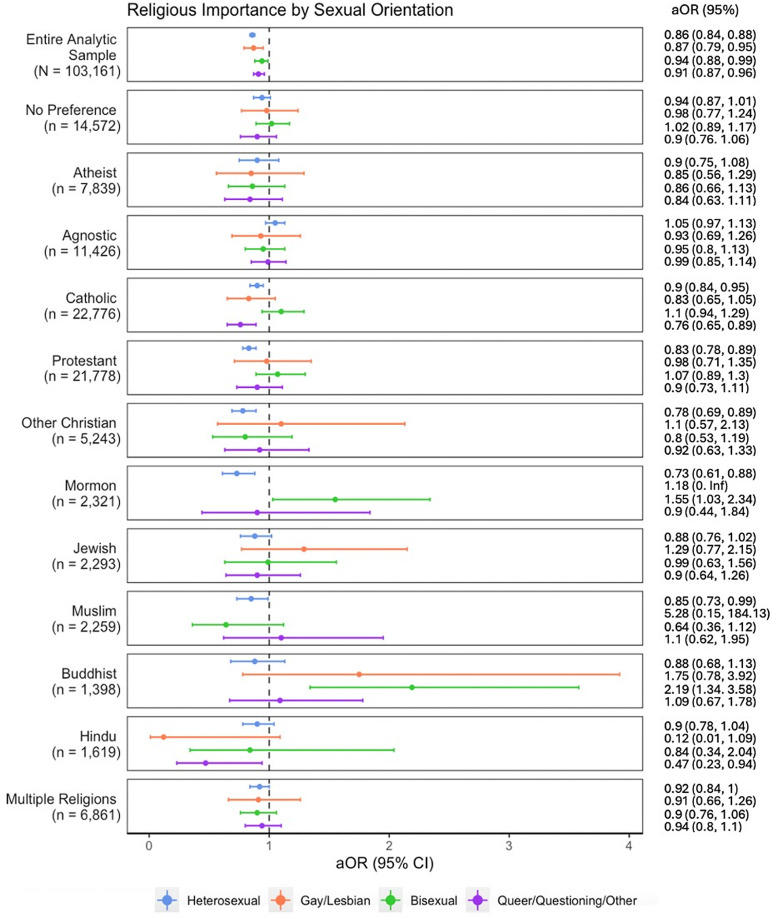
Associations between religious importance and depression by sexual orientation, stratified by religious affiliation, Healthy Minds Study, 2020-2021.

In Fig 1, we present the associations between religious importance and depression in the entire sample, stratified by sexual minority group and by religious affiliation. We found that the associations between religious importance and depression varied across sexual minority groups and religious affiliations, although the differences across sexual minority groups were only statistically significant among Catholic students. As expected, for those reporting no preference/atheist/ agnostic, religious importance was not associated with depression for any sexual minority group. Among Christian students, higher religious importance was associated with lower odds of depression. This was true among Protestant, Catholic, Other Christian, and Mormon affiliations. For all these affiliations, the protective effects were only evident for heterosexual students, but not for sexual minority groups, except for Catholic queer/questioning/other students. Higher religious importance was also associated with lower odds of depression among Muslim heterosexual students and queer/questioning/other Hindu students. Further, higher religious importance was associated with greater odds of depression for bisexual Buddhist students and bisexual Mormon students.

## Discussion

In this study we found a complex portrait of religiousness, sexual orientation, and depression among students in U.S. higher education. To our knowledge, this is among the largest-sampled studies to examine the interaction between religious importance, sexual orientation identity, and depression in a religiously, culturally, and sexually diverse sample of emerging adults in U.S. higher education institutions. We found that religious affiliation with most of the major world religions was associated with lower odds of depression, but this was not necessarily true for all affiliations, depending on comparison groups. However, among sexual minority students, the protective effect of religious affiliation was weaker and sometimes not statistically significant. Our findings comport with a study conducted by Klundt and colleagues [52], who found that sexual minority college students at a conversative university reported worse mental health outcomes and well-being than their heterosexual counterparts. Similar to our findings, their study showed that religiousness was protective for both heterosexual and sexual minority students, although weaker for the latter.

Greater religious importance overall appeared to be associated with lower odds of depression. It is important to note that even for atheists, the point estimates suggested religious importance was protective against depression across all sexual orientations, though not statistically significant. It may seem odd that people who reported being atheists also found their religious beliefs to be important. While some atheists see themselves as not having any religious beliefs, there are some who view atheism as an organizing worldview that they find important [53]. Again, the association between religious importance and depression depended on sexual minority status and religious affiliation. For the major Christian religious affiliations (e.g., Protestant, Catholic), the protective effect of religious importance was only evident for heterosexual individuals and not for any of the sexual minority groups. Among Hindu and Catholic religions, greater religious importance was associated with lower odds of depression for heterosexual students, but also queer/questioning/other students.

Religion can be a source of strength and happiness for sexual minorities [54]. However, studies have also shown that religiousness may be only slightly protective, non-significant, or even harmful for sexual minorities. For instance, religious belief systems can instill a sense of hope, meaning, and purpose in life [14,55]; yet, some of these same religious belief systems may condemn homosexuality and therefore foster guilt and shame in sexual minorities. Some religious sexual minorities may even go so far as to engage in sexual orientation change efforts, which have been linked to depression and suicide [37]. Religiousness may be especially protective by imparting a sense of belonging and social connectedness, while granting access to social support [15,46,56]. But these health-promoting aspects may be attenuated for sexual minorities who encounter negative social interactions (discrimination and condemnation), thereby leading to feelings of isolation, rejection, thwarted belongingness, and minority stress. Klundt and colleagues [52] found that a major predictor of poor mental health (higher depression, anxiety, distress, and suicidality) and lower quality of life among sexual minority students was the concern of not being accepted by others at their conservative university. In our study, the finding that religious importance was not significantly protective for sexual minority students among certain religious affiliations may be attributable to how tolerant or accepting a given religion is toward same-sex relationships.

Further, the moderating effect of sexual orientation remains complicated across affiliations, showing considerable nuances at the intersections of identity. For instance, we found among students who were affiliated with Catholicism and identified as queer/questioning/other sexual orientation, religious importance was associated with significantly lower odds of depression. While there is currently no working hypothesis on why we see protective effects of religious importance in this particular sexual minority group (and not other sexual minority groups) in a religion that does not officially affirm same-sex relationships, we do note that surveys from the Pew Research Center suggest 85% of young adult Catholics believe ‘homosexuality should be accepted by society’ [57], though this level of support/acceptance was not as high among Protestants or Mormons [58,59]. It is unclear if these public opinions may have influenced the associations we found for certain sexual minority groups. We also found that among Buddhist and Mormon religions, greater religious importance was associated with higher odds of depression for bisexual individuals. Emerging research shows that individuals who identify as bisexual often report worse mental health outcomes compared with lesbian/gay people [60–63]; however, more research is needed to understand the risk and resilience factors for religious bisexual individuals (e.g., feeling ‘erased’ or invalidated by both religious and LGBT+ communities). Why religious importance would be associated with greater odds of depression among bisexual Buddhist students is another area that requires future research; however, it is worth mentioning that there are multiple traditions of Buddhism (e.g., Mahayana, Theravada, Vajrayana) that have different stances on homosexuality, and Buddhist religious culture may overlap or syncretize with more conservative socio-cultural norms in Asia.

### Limitations

The present study findings must be interpreted bearing in mind several limitations. First, in terms of design, the study was cross-sectional, and therefore did not allow for any causal inferences. Longitudinal studies can help ascertain whether heterosexual depressed individuals are more or less likely to turn to religion for help (e.g., coping) than sexual minority individuals.

Second, in terms of measurement, the categorization of religious affiliations may have been somewhat arbitrary. We followed the Pew Research Center classification system, which has been developed for large survey studies of the general population in the US. However, even so, many students appeared to have complex religious beliefs that were not easy to classify, and categories often conflated distinct world religions, resulting in the formation of categories that were highly heterogenous and difficult to interpret. For example, having multiple religious affiliations was coded in a way that captures individuals who selected both Mormon and Protestant, but also individuals who selected Atheism and Agnosticism. Another example is that ‘Other World Religion’ arbitrarily contained affiliations such as Sikhism, Shintoism, Punjabi, and numerous other religions that are distinct but too few to statistically account for. Future research should explore whether religious importance is related to depression among these other world religions and among people who select more than one affiliation.

Third, we lacked measures of various aspects of religiousness, such as worship service attendance, theological beliefs (e.g., how affirming the religion/denomination is), or private religious practices (e.g., prayer, scripture reading). Future studies should longitudinally explore specific aspects of religiousness and their effects on depression over time for various sexual minority groups. The measures in the HMS were largely cognitive, and the behavioral aspects of religiousness may be even more impactful on mental health. For example, participating in extracurricular religious activities may be an important marker of religious commitment and religious community integration, protecting against mental health problems. However, the effects of these extracurricular activities for sexual minority students could in some instances be associated with suicidal ideation, depending on the university [39].

Fourth, the sexual minority variable overlooked the multiple complex and sometimes contradictory nature of sexuality, which includes components such as identity, behavior, and desire. It is possible that people may have been reluctant to disclose their sexual orientation on surveys, especially highly conservative religious students. For instance, the gay/lesbian group was especially small among Mormon students, and it is possible this small group may have been affected by issues relating to public outness, identity conflicts, and internalized homonegativity. Overall, the HMS sample was large but still not large enough to detect all subgroup effects across sexual minority groups and religious affiliations.

Finally, in terms of sampling, the findings can only reflect students in higher education who were willing to complete the survey. The response rate was relatively low but expected for a survey of this nature. We used survey weights to account for non-response; however, sampling bias remains a concern. Regarding representativeness, future studies can explore how contexts shape the associations between religiousness and depression across sexual orientations. The intersection of sexual minority status, religious affiliation, and geographic location (e.g., West vs. South; urban vs. rural) may reveal a complex picture of mental health risk.

### Implications

Our findings suggest that religiousness is an important aspect of life for many students in higher education, and that being religiously affiliated and perceiving religion to be important are associated with lower odds of depression. However, certain aspects of religiousness may be less protective for sexual minority students. Clinicians, clergy, and other gatekeepers serving student populations should elicit information about religious affiliation and religious importance, as well as any potential qualms that may arise at the intersection of religiousness and sexual orientation. The moderated effect we found in our study may be traced to religious contexts where heterosexual and sexual minority students are treated differently; namely, sexual minorities may be more likely to have negative experiences in religious spaces [36]. As such, sexual minorities may experience barriers to believing (e.g., heteronormative doctrines), bonding (e.g., barriers to pursuing full membership or engagement in religious traditions), behaving (e.g., religious proscriptions to same-sex sexual behavior), and belonging (e.g., discrimination and rejection from conservative co-religionists) that are not experienced by their heterosexual counterparts [31]. Thus, these additional barriers may make the benefits of religiousness less unambiguously positive [33]. Further, these barriers may even lead many sexual minorities to experience religious/spiritual struggles [30] and/or de-identify with religion, making sexual minorities even less likely to access the benefits of religion. Holding space for students to explore, negotiate, and self-author their religious and sexual identities may prove beneficial for mental health. Additionally, heterosexism and homophobia can impart social stress and create barriers to health promoting resources, thereby impacting the mental health and wellness of sexual minorities. Thus, practitioners, policy makers, and researchers should coordinate public health efforts to reduce and ultimately eliminate the heterosexism and homophobia that pervades structures, institutions, and cultures.

### Conclusion

Religious affiliation and religious importance were associated with lower odds of depression for both heterosexual and sexual minority students, but the associations were generally less strong for sexual minority students and varied depending on religious affiliation.

## Supporting information

S1 Fig Sexual orientation groups across religious affiliations. (DOCX)

S2 Fig Prevalence of depression by religious affiliation. (DOCX)

S3 Fig Mean depression scores across religious affiliations. (DOCX)

S4 Fig Religious importance by sexual orientation. (DOCX)

S1 Table Descriptive statistics. (DOCX)

S2 Table Multivariable logistic regression models showing associations between religious affiliation and depression over the past two weeks, Healthy Minds Survey 2020-2021 (N=103,161). (DOCX)
