# Correction: Religiousness, sexual orientation, and depression among emerging adults in U.S. higher education: Findings from the Healthy Minds Study

**DOI:** 10.1371/journal.pmen.0000521

**Published:** 2025-12-19

**Authors:** Hans Oh, G. Tyler Lefevor, Edward B. Davis, Anna Zhu, Yaofang Hu, Trevor A. Pickering, Ai Koyanagi, Lee Smith

An additional affiliation is missing for the first author. Hans Oh is also affiliated with the School of Public Health, University of California, Berkeley, Berkeley, California, United States of America.
