# Supplementary material for: Religiousness, sexual orientation, and depression among emerging adults in U.S. higher education: Findings from the Healthy Minds Study
Source: PLOS Ment Health. 2025 Mar 26;2(3):e0000004. doi: 10.1371/journal.pmen.0000004 (PMC12798255; doi:10.1371/journal.pmen.0000004)
Supplement: S1 Fig — (DOCX) [file pmen.0000004.s001.docx]

**S1 Fig. Sexual orientation groups across religious affiliations**
