# Supplementary figures and images for: Religiousness, sexual orientation, and depression among emerging adults in U.S. higher education: Findings from the Healthy Minds Study
Source: PLOS Ment Health. 2025 Mar 26;2(3):e0000004. doi: 10.1371/journal.pmen.0000004 (PMC12798255; doi:10.1371/journal.pmen.0000004)

**S2 Fig. Prevalence of depression by religious affiliation**


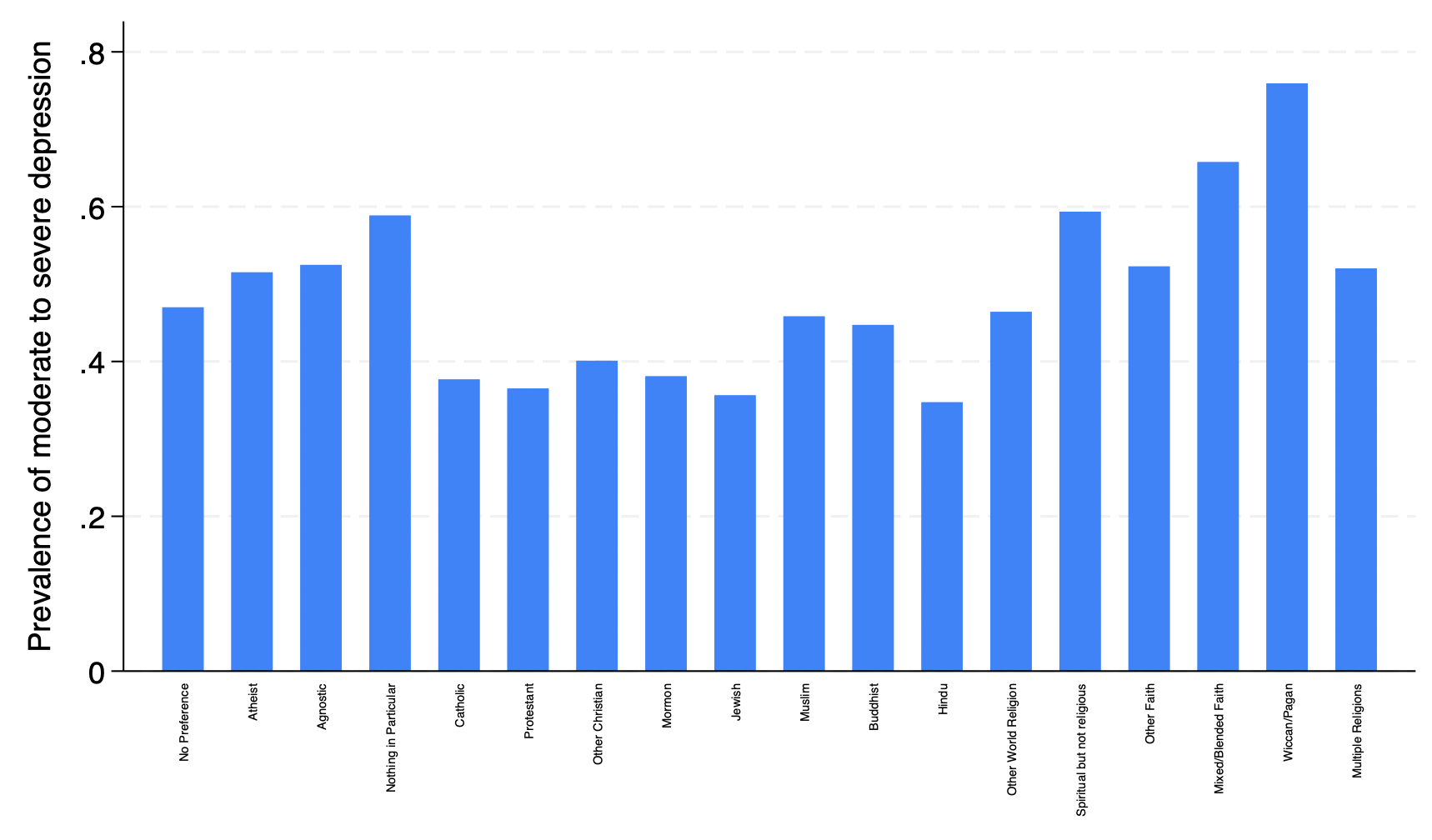

Supplement: S2 Fig — (DOCX) [file pmen.0000004.s002.docx]
