# Supplementary material for: Religiousness, sexual orientation, and depression among emerging adults in U.S. higher education: Findings from the Healthy Minds Study
Source: PLOS Ment Health. 2025 Mar 26;2(3):e0000004. doi: 10.1371/journal.pmen.0000004 (PMC12798255; doi:10.1371/journal.pmen.0000004)
Supplement: S1 Table — (DOCX) [file pmen.0000004.s005.docx]

| **S5 Table. Descriptive statistics** | | | |
| --- | --- | --- | --- |
|  | Depression (N=44,670) | No Depression (N=58,491) | Total (N=103,161) |
|  | N,% | N,% | N,% |
| **Religious Affiliation** |  |  |  |
| Unaffiliated | 17,005 (37.31%) | 17,054 (28.29%) | 34,059 (32.15%) |
| Christian Religion | 19,510 (45.41%) | 32,608 (58.69%) | 52,118 (53.01%) |
| Non-Christian Religion | 4,471 (9.36%) | 5,449 (7.76%) | 9,920 (8.45%) |
| Multiple Religions | 3,571 (7.68%) | 3,290 (5.03%) | 6,861 (6.16%) |
| Other Religion | 113 (0.23%) | 90 (0.23%) | 203 (0.23%) |
|  |  |  |  |
| **Religious importance** (mean, 95% CI) | 2.970 (2.90, 3.04) | 3.339 (3.249, 3.49) | 3.18 (3.10, 3.26) |
|  |  |  |  |
| **Sexual orientation** |  |  |  |
| Heterosexual | 28,529 (64.93%) | 48,976 (84.90%) | 77,505 (76.36%) |
| Sexual Minority | 16,141 (35.07%) | 9,515 (15.10%) | 25,656 (23.64%) |
|  |  |  |  |
| **Age** (mean, 95% CI) | 21.031 (20.91, 21.15) | 21.243 (21.058, 21.429) | 21.15 (21.00, 21.30) |
|  |  |  |  |
| **Gender** |  |  |  |
| Man | 9,830 (32.47%) | 18,534 (44.93%) | 28,364 (39.60%) |
| Woman | 32,268 (61.87%) | 39,053 (53.62%) | 71,321 (57.15%) |
| Other | 2,572 (5.66%) | 904 (1.45%) | 3,476 (3.25%) |
|  |  |  |  |
| **Race/ethnicity** |  |  |  |
| White | 27,181 (60.18%) | 36,341 (61.48%) | 63,522 (60.92%) |
| Black | 3,743 (9.75%) | 5,020 (11.67%) | 8,763 (10.85%) |
| Asian/Pacific Islander | 4,801 (8.79%) | 7,516 (9.64%) | 12,317 (9.28%) |
| Latinx/Hispanic | 3,316 (8.66%) | 3,724 (7.33%) | 7,040 (7.90%) |
| Multiracial | 4,834 (11.10%) | 5,020 (8.59%) | 9,854 (9.66%) |
| Other | 795 (1.52%) | 870 (1.28%) | 1,665 (1.38%) |
