# Supplementary material for: Religiousness, sexual orientation, and depression among emerging adults in U.S. higher education: Findings from the Healthy Minds Study
Source: PLOS Ment Health. 2025 Mar 26;2(3):e0000004. doi: 10.1371/journal.pmen.0000004 (PMC12798255; doi:10.1371/journal.pmen.0000004)
Supplement: S2 Table — (DOCX) [file pmen.0000004.s006.docx]

| **S6 Table. Multivariable logistic regression models showing associations between religious affiliation and depression over the past two weeks, Healthy Minds Survey 2020-2021 (N=103,161)** | | | | |
| --- | --- | --- | --- | --- |
|  | Heterosexual | | Sexual minority | |
| Religious affiliations | aOR (95% CI) | P-Value | aOR (95% CI) | P-Value |
| Unaffiliated | 1.00 |  | 1.00 |  |
| Christian | 0.69 (0.64, 0.74) | <0.001 | 0.77 (0.7, 0.85) | <0.001 |
| Non-Christian Religion | 0.89 (0.81, 0.98) | 0.016 | 1.16 (1.00, 1.35) | 0.045 |
| Multiple Religions | 1.08 (0.97, 1.19) | 0.159 | 1.13 (1.01, 1.26) | 0.030 |
| Don’t Know | 0.76 (0.41, 1.4) | 0.371 | 1.12 (0.49, 2.56) | 0.786 |
| Adjusted for age, gender, race/ethnicity | | | | |
